# Genetic polymorphisms of 3′-untranslated region of *SULT1A1* and their impact on tamoxifen metabolism and efficacy

**DOI:** 10.1007/s10549-018-4923-7

**Published:** 2018-08-17

**Authors:** A. B. Sanchez-Spitman, V. O. Dezentjé, J. J. Swen, D. J. A. R. Moes, H. Gelderblom, Henk-Jan Guchelaar

**Affiliations:** 1 Department of Clinical Pharmacy & Toxicology, Leiden University Medical Center, Albinusdreef 2, 2300 RC Leiden, The Netherlands; 2 Leiden Network for Personalised Therapeutics, Leiden University Medical Center, Leiden, The Netherlands; 3 Department of Medical Oncology, Netherlands Cancer Institute/Antoni van Leeuwenhoek, Amsterdam, The Netherlands; 4 Department of Medical Oncology, Leiden University Medical Center, Leiden, The Netherlands

**Keywords:** Tamoxifen, Endoxifen, Rs6839, Rs1042157, SULT1A1

## Abstract

**Purpose:**

Tamoxifen has a wide inter-variability. Recently, two SNPs in the 3′-untranslated region (UTR) of the *SULT1A1* gene, rs6839 and rs1042157, have been associated with decreased SULT1A1 activity. The aim of this study is to investigate the role of the rs6839 and rs1042157 on tamoxifen metabolism and relapse-free survival (RFS) in women diagnosed with early-breast cancer receiving tamoxifen.

**Methods:**

Samples from 667 patients collected in the CYPTAM study (NTR1509) were used for genotyping (*CYP2D6*, SULT1A1 rs6839 and rs1042157) and measurements of tamoxifen and metabolites. Patients were categorized in three groups depending on the decreased SULT1A1 activity due to rs6839 and rs1042157: low activity group (rs6839 (GG) and rs1042157 (TT)); high activity group (rs6839 (AA) and rs1042157 (CC)); and medium activity group (all the other combinations of rs6839 and rs1042157). Associations between SULT1A1 phenotypes and clinical outcome (RFS) were explored.

**Results:**

In the low *SULT1A1* activity group, higher endoxifen and 4-hydroxy-tamoxifen concentrations were found, compared to the medium and high activity group (endoxifen: 31.23 vs. 30.51 vs. 27.00, *p* value: 0.016; 4-hydroxy-tamoxifen: 5.55 vs. 5.27 vs. 4.94, *p* value:0.05). In terms of relapse, the low activity group had a borderline better outcome compared to the medium and high *SULT1A1* activity group (adjusted Hazard ratio: 0.297; 95% CI 0.088–1.000; *p* value: 0.05).

**Conclusion:**

Our results suggested that rs6839 and rs1042157 SNPs have a minor effect on the concentrations and metabolic ratios of tamoxifen and its metabolites, and RFS in women receiving adjuvant tamoxifen.

**Electronic supplementary material:**

The online version of this article (10.1007/s10549-018-4923-7) contains supplementary material, which is available to authorized users.

## Introduction

**Fig. 1:**
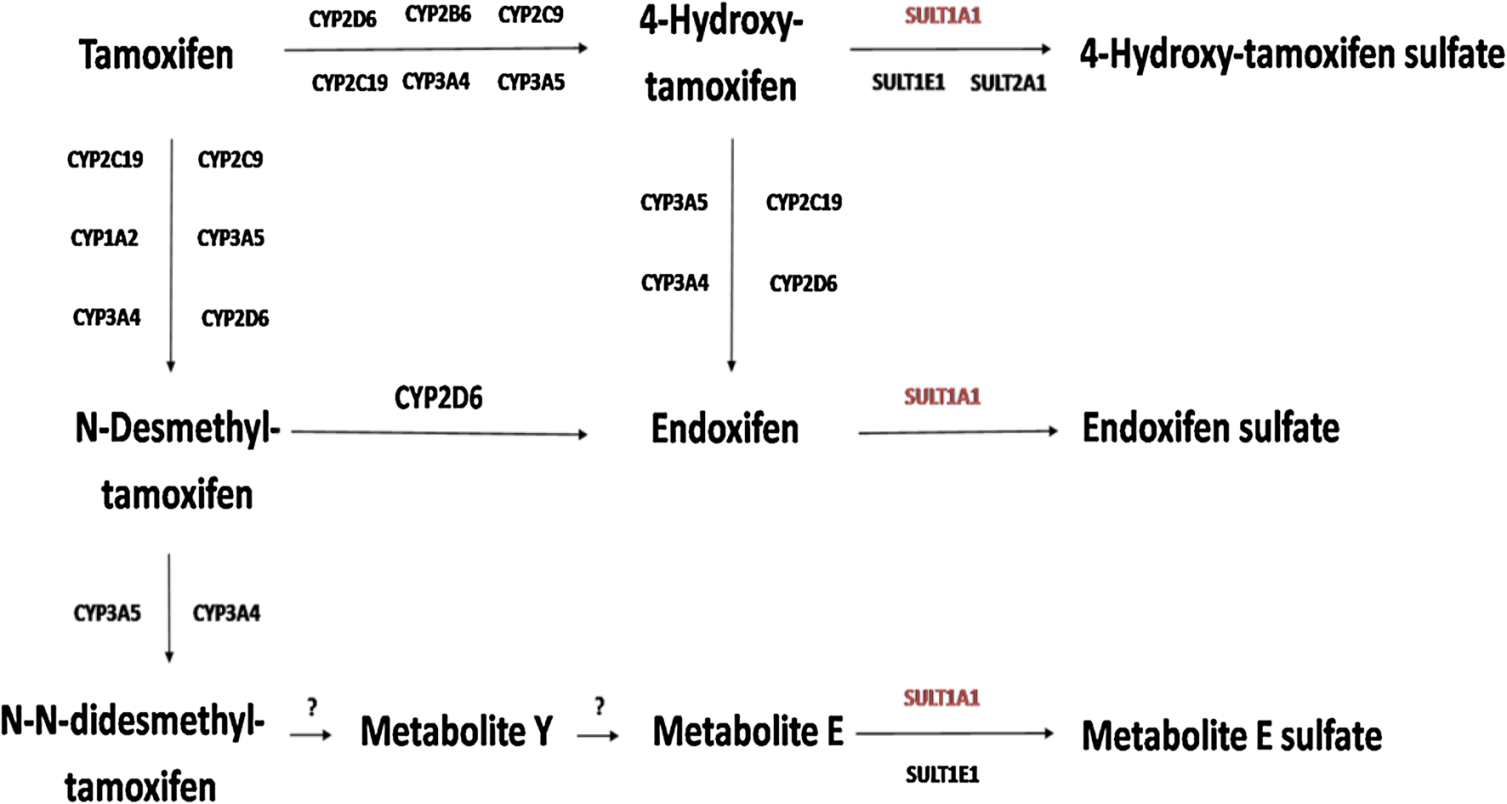
Tamoxifen metabolism

Tamoxifen is commonly used as adjuvant endocrine therapy to treat patients diagnosed with breast cancer [1, 2]. Being a prodrug, tamoxifen is bioactivated by several cytochrome P-450 enzymes to its primary metabolites, 4-hydroxy-tamoxifen, and *N*-desmethyl-tamoxifen (NDM-tamoxifen). Thereafter, conversion into endoxifen takes place (Fig. 1), mainly controlled by CYP2D6, among other enzymes. Around 92% of tamoxifen metabolism accounts for the biotransformation of tamoxifen into NDM-tamoxifen, whereas the conversion of tamoxifen into 4-hydroxy-tamoxifen only represents 7% [3].

Both endoxifen and 4-hydroxy-tamoxifen have equal affinity for the estrogen receptor α [4], but endoxifen is considered the most clinically relevant tamoxifen metabolite, since it is found in 5–10 times higher concentrations than 4-hydroxy-tamoxifen [5]. While CYP2D6 is the rate-limiting enzyme in tamoxifen metabolism, it cannot fully explain the inter-patient variability of tamoxifen metabolism [6]. Other genetic polymorphisms in different enzymes than CYP2D6 have been suggested to influence tamoxifen metabolism as well [7].

Sulfotransferases (SULTs) are classified as phase II enzymes involved in the biotransformation of a variety of drugs [7, 8]. By adding a sulfonyl group to xeno- and endobiotics, more hydrophilic molecules are obtained facilitating their renal excretion [8, 9]. SULT1A1 is the most expressed isoform of the SULT enzymes in the human liver [10, 11]. In tamoxifen metabolism, SULT1A1 mainly catalyzes the transformation of 4-hydroxy-tamoxifen into inactive 4-hydroxy-tamoxifen sulfate and endoxifen into inactive endoxifen sulfate (Fig. 1). In addition, SULT1A1 is also involved in the inactivation of NDM-tamoxifen, after several consecutive reactions, into Metabolite E sulfate [3, 9, 12, 13].

Several *SULT1A1* Single-Nucleotide Polymorphisms (SNPs) have been described and found associated with clinical outcome in tamoxifen-treated patients. Nowell [14] and Wegman [15] reported that SULT1A1*2/*2 carriers had worse outcome in breast cancer patients treated with tamoxifen compared to both homozygous and heterozygous SULT1A1*1 carriers. However, studies performed later did not reproduce these results, since no significant associations were found [16–18]. Consequently, the effect of *SULT1A1* and clinical outcome among tamoxifen-treated patients is still unclear.

*SULT1A1* genetic variation and its influence on tamoxifen and its metabolites concentrations and metabolic ratios (MR) have been described. While Jin [19] and Fernandez-Santander [20] showed no association between *SULT1A1* genotypes and tamoxifen and its metabolites concentrations, Gjerde and colleagues found an association between *SULT1A1* genotype and the metabolic ratios (MR) of NDM-tamoxifen/tamoxifen (Fig. 1) [21].

In the same manner, copy number variation in *SULT1A1* has been described as a prominent contributor to the inter-variability of SULT1A1 enzymatic activity [22]. Hebbring and colleagues reported an *in vitro* association between CNV and SULT1A1 enzyme activity. The role of *SULT1A1* CNVs in tamoxifen efficacy has also been examined, but no significant relationship after 14 years of follow-up between disease-free survival and *SULT1A1* CNVs was found [22]. However, this result might be explained by ethnic differences in the enrolled women, who were primarily Caucasian. Indeed, *SULT1A1* CNV is most frequently seen in African-American individuals, but infrequently occurs in other ethnicities [22].

Recently, two other *SULT1A1* SNPs, rs6839 and rs1042157, have been identified and characterized in the 3′-untranslated region (UTR) of the *SULT1A1* gene [23]. According to the authors, both SNPs are in linkage disequilibrium (*D*′ = 0.83) and associated with decreased activity of the SULT1A1 enzymatic activity. To date, only two studies have analyzed the effect of both SNPs and cancer risk [24, 25].

To the best of our knowledge, the role of rs6839 and rs1042157 in tamoxifen metabolism and RFS has not yet been examined. Therefore, the aim of the current study is to explore the role of the rs6839 and rs1042157 SNPs on tamoxifen pharmacokinetics and RFS in the CYPTAM cohort of women with early breast cancer using adjuvant tamoxifen [26, 27].

## Methods

Study design and objectives: effect of 3′-UTR of *SULT1A1* SNPs on tamoxifen metabolism and clinical outcome.

The CYPTAM study (NTR1509) is a completed prospective clinical study carried out in Belgium and The Netherlands [26]. The aim of this clinical study was to investigate CYP2D6 predicted phenotypes and endoxifen serum concentrations with clinical outcome (relapse-free and disease-free survival, and overall survival). Briefly, women using tamoxifen at a daily dose of 20 mg as adjuvant endocrine therapy for early breast cancer were asked to participate in this multicenter study. The study protocol of the CYPTAM study was approved by The Medical Ethical Committee of the Leiden University Medical Center (The Netherlands). Written informed consent was obtained from all of the included patients. Pregnancy, breast feeding, and previous malignancy were considered exclusion criteria, with the exception of appropriately treated patients with in-situ cervix carcinoma and basal cell carcinoma. After receiving tamoxifen for a minimum of two months, whole blood and serum samples were collected for genotyping and determination of tamoxifen and its metabolites (NDM-tamoxifen, 4-hydroxy-tamoxifen and endoxifen), respectively.

To investigate the role of rs6839 and rs1042157 SNPs, serum and whole blood samples and clinical data and follow-up from women enrolled in the CYPTAM were readily available for analysis. Since both rs6839 and rs1042157 SNPs are in linkage disequilibrium, groups were required in order to understand the combined effect of both SNPs on tamoxifen metabolism and efficacy. Therefore, three different groups were made according to the known effect of rs6839 and rs1042157 on SULT1A1 enzyme activity. These groups were defined as low, medium, and high SULT1A1 activity groups, as follows: low activity group was defined as the combination of rs6839 (GG) and rs1042157 (TT); high activity group was compound by rs6839 (AA) and rs1042157 (CC); medium activity group was formed by the following combinations: rs6839 (AG) and rs1042157 (CC); rs6839 (AA) and rs1042157 (CT); rs6839 (AG) and rs1042157 (CT); rs6839 (GG) and rs1042157 (CT); rs6839 (AA) and rs1042157 (TT); and rs6839 (AG) and rs1042157 (TT).

The first objective of this pharmacogenetic study was to compare the combined effect of both SNPs on tamoxifen metabolism by comparing differences in endoxifen concentrations and metabolic ratios of tamoxifen and its metabolites (NDM-tamoxifen, 4-hydroxy-tamoxifen, and endoxifen) across the different groups. The secondary objective of this research was to investigate the impact of the 3′- UTR *SULT1A1* SNPs groups on tamoxifen efficacy. In the CYPTAM study, the primary endpoint was relapse-free survival (RFS), defined as the time from study enrolment until loco-regional recurrence, second breast cancer, or distant recurrence. If patients switched to an aromatase inhibitor, patients were censored at the time of tamoxifen discontinuation [26].

### Tamoxifen and its metabolites measurements

In order to ensure tamoxifen and metabolite steady-state concentrations, a minimum of two-month treatment with tamoxifen was required before sampling. To adequately assess tamoxifen and its metabolites trough levels, samples were collected at least twelve hours after the last tamoxifen intake.

Concentrations were determined using high-performance liquid chromatography-tandem mass spectrometry (HPLC-MS/MS). The bioanalytical assay was developed and validated by the laboratory of Clinical Pharmacy and Toxicology Department at Leiden University Medical Center, and it is a method comparable to another method already reported [28].

### Genotyping: CYP2D6, rs6839, and rs1042157

CYP2D6 genotyping was performed with Amplichip CYP450 test (Roche Diagnostic, Indianapolis, US) to evaluate the major CYP2D6 alleles in DNA previously retrieved from the CYPTAM patients. More detailed information regarding the CYP2D6 genotypes is described elsewhere [29, 30]. Genotype analysis for rs6839 and rs1042157 was performed using Pyrosequencing (Qiagen, Venlo, The Netherlands) following standard procedures and the instructions of the manufacturer.

### Statistical analysis

To test linkage disequilibrium between both rs6839 and rs1042157, D′ was calculated with Chi-square statistics (*χ*^2^). Metabolic ratios were defined as concentration of substrate divided by metabolite concentration. ANOVA test was used to compare mean concentration levels and metabolic ratios of tamoxifen and its metabolites (NDM-tamoxifen, 4-hydroy-tamoxifen and endoxifen) between the low, medium, and high *SULT1A1* activity groups. Multiple linear regression analysis was used to analyze the contributions of rs6839 and rs1042157. By using the base model in which the CYP2D6 status only partly contributes to explaining the total variability of concentrations and metabolic ratios of tamoxifen, endoxifen, 4-hydroxy-tamoxifen, and NDM-tamoxifen, these 3′-UTR *SULT1A1* rs6839 and rs1042157 SNPs were added to the model to investigate their effects on the total variance.

Cox regression analysis was performed to assess whether RFS varied according to the different baseline characteristics across all the groups. If in the univariable analysis, a covariable had a *p* value below 0.1, this covariable was fitted in the multivariable model. Due to their clinical importance for the survival outcome in breast cancer patients, tumor and nodal stage, Her2 receptor status, and histological grade and classification were also included in the multivariable analysis, regardless of the results in the univariable analysis. Kaplan–Meier method was used to estimate the distributions of RFS, whereas a log-rank test was performed to compare the clinical outcome with genetic 3′-UTR *SULT1A1* rs6839 and rs1042157 SNPs. Statistical analyses were assessed with IBM SPSS for Windows, Version 23.0. In all cases, *p* values below 0.05 were considered statistically significant.

## Results

### Study population

In the CYPTAM study, 667 women were included in 25 Dutch and Belgian hospitals. More detailed clinical characteristics of the included patients in the core CYPTAM study are reported elsewhere [30, 31].

For the purpose of this pharmacogenetic study, three groups with low, medium, and high SULT1A1 activity groups were made. At enrolment, all groups of patients were comparable regarding mean age, tumor and nodal stage, histologic grade and classification, HER2 and progesterone receptor status, type of main surgery (mastectomy or breast conserving surgery) and axillar surgery (sentinel node procedure only or axillary lymph node dissection), adjuvant radiotherapy and chemotherapy, and treatment with trastuzumab (*p* value > 0.05). An overview of the baseline characteristics of the enrolled patients by the three groups is listed in Table 1.

**Table 1:** Baseline characteristics of the CYPTAM patients by 3’ UTR SULT1A1 high, medium, and low activity groups

		3′ UTR SULT1A1 rs6839 and rs1042157 SNPs groups						*p* value
		High activity group (*N* = 231)		Medium activity group (*N* = 324)		Low activity group (*N* = 105)		
		*N*	(%)	*N*	(%)	*N*	(%)	
Age at enrolment	Mean in years (SD)	56.2	11.2	56.9	11.4	54.6	9.8	0.155
Tumor stage	T1	121	52.4	170	52.5	58	55.2	0.936
	T2	96	41.6	137	42.3	41	39.0	
	T3/T4	12	5.2	12	3.7	4	3.8	
	Not specified	2	0.9	5	1.5	2	1.9	
Nodal stage	N0	110	47.6	158	48.8	45	42.9	0.719
	N1	92	39.8	129	39.8	43	41.0	
	N2	19	8.2	27	8.3	10	9.5	
	N3	10	4.3	8	2.5	6	5.7	
	Not specified	0	0.0	2	0.6	1	1.0	
Histological classification	Ductal adenocarcinoma	178	77.1	248	76.5	78	74.3	0.738
	Lobular adenocarcinoma	35	15.2	42	13.0	14	13.3	
	Other	18	7.8	32	9.9	12	11.4	
	Not specified	0	0.0	2	0.6	1	1.0	
Histological grade	G1	36	15.6	42	13.0	16	15.2	0.702
	G2	124	53.7	189	58.3	61	58.1	
	G3	70	30.3	89	27.5	26	24.8	
	Not specified	1	0.4	4	1.2	2	1.9	
Progesterone receptor status	Positive	186	80.5	256	79.0	85	81.0	0.973
	Negative	42	18.2	63	19.4	18	17.1	
	Not specified	3	1.3	5	1.5	2	1.9	
HER2 receptor status	0	135	58.4	209	64.5	58	55.2	0.449
	1+	68	29.4	71	21.9	28	26.7	
	2+	11	4.8	17	5.2	7	6.7	
	3+	17	7.4	25	7.7	11	10.5	
	Not specified	0	0.0	2	0.6	1	1.0	
FISH	Positive (amplification)	17	7.4	29	9.0	11	10.5	0.584
	Negative	214	92.6	293	90.4	93	88.6	
	Not specified	0	0.0	2	0.6	1	1.0	
Surgery	Mastectomy	116	50.2	142	43.8	47	44.8	0.347
	Breast conserving	114	49.4	180	55.6	56	53.3	
	Not specified	1	0.4	2	0.6	2	1.9	
Surgery axilla	Sentinel node procedure only	110	47.6	164	50.6	55	52.4	0.517
	Axillary lymph node dissection	120	51.9	158	48.8	48	45.7	
	Not specified	1	0.4	2	0.6	2	1.9	
Adjuvant radiotherapy	Yes	156	67.5	231	71.3	71	67.6	0.546
	No	75	32.5	91	28.1	33	31.4	
	Not specified	0	0.0	2	0.6	1	1.0	
Adjuvant chemotherapy	Yes	137	59.3	198	61.1	66	62.9	0.664
	No	94	40.7	124	38.3	38	36.2	
	Not specified	0	0.0	2	0.6	1	1.0	
Trastuzumab therapy	Yes	19	8.2	28	8.6	10	9.5	0.442
	No	212	91.8	291	89.8	94	89.5	
	Not specified	0	0.0	5	1.5	1	1.0	

*3′UTR* 3′ Untranslated region; *SD* standard deviation

### Genotype distributions: rs6839 and rs1042157 SNPs

Genotype distribution for rs1042157 was consistent with Hardy–Weinberg equilibrium (*χ*2 = 2.98, *p* = 0.084), while for rs6839 it was found not to be in Hardy–Weinberg equilibrium (*χ*2 = 13.44, *p* = 0.00025). However, genotype frequencies of rs6839 were similar to allelic frequencies reported previously for the Caucasian population and described on the National Center for Biotechnology Information website (NCBI, http://www.ncbi.nlm.nih.gov). Linkage Disequilibrium was analyzed for both 3′-UTR SULT1A1 variants and a significantly strong association was found for rs6839 and rs1042157 (*D*′ = 0.74, *p* < 0.0001). The variant allele frequencies of rs6839 and 1,042,157 are described in Supplementary Table 1.

### Association between tamoxifen and its metabolites and 3′-UTR *SULT1A1* groups

**Fig. 2:**
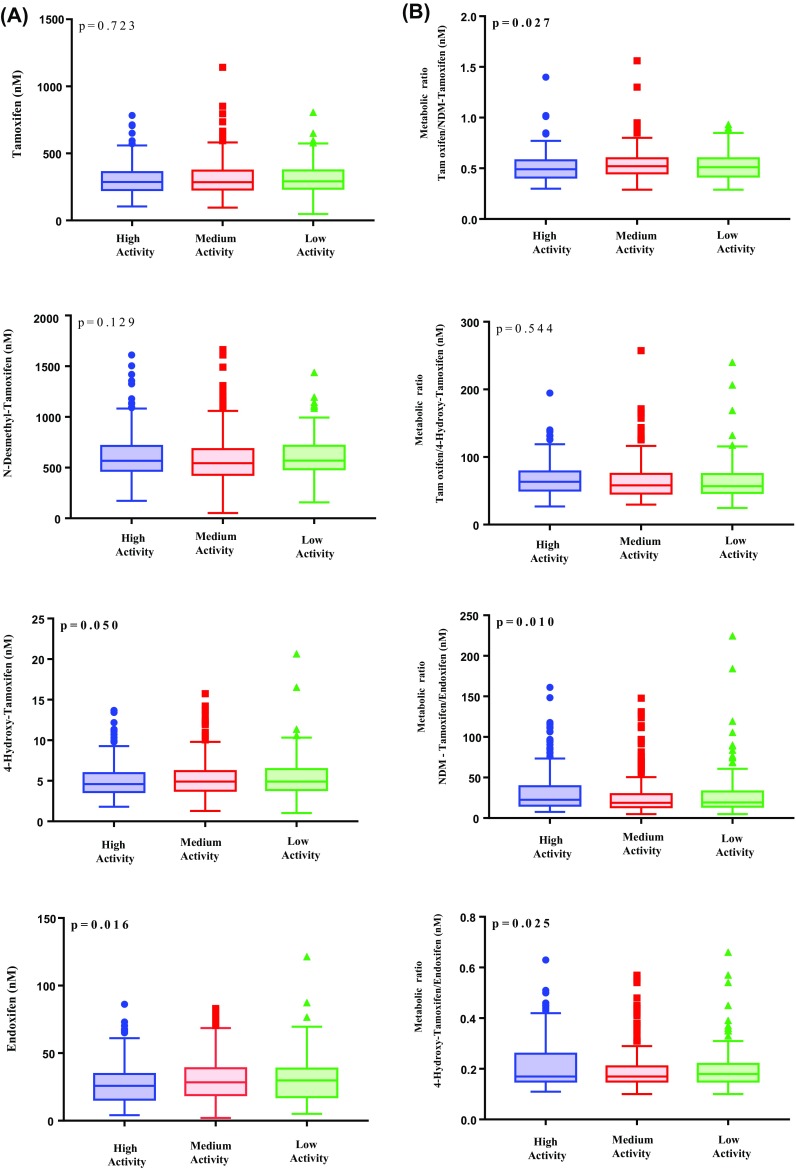
Association with tamoxifen and its metabolites. **a** Association of tamoxifen, endoxifen, 4-hydroxy-tamoxifen, and NDM-tamoxifen concentration levels by high, medium, and low SULT1A1 enzyme activity groups. **b** Association of tamoxifen, endoxifen, 4-hydroxy-tamoxifen, and NDM-tamoxifen metabolic ratios by high, medium, and low SULT1A1 enzyme activity groups

The mean concentration levels of tamoxifen and NDM-tamoxifen across the 3′-UTR *SULT1A1* groups did not significantly differ (*p* > 0.05). In contrast, endoxifen and 4-hydroxy-tamoxifen mean concentrations in the low activity group were statistically significantly higher, compared to the other groups (endoxifen: *p* value = 0.016; 4-hydroxy-tamoxifen: *p* value = 0.050). Figure 2 shows the associations comparing low, medium, and high activity groups regarding the mean concentrations and metabolic ratios of tamoxifen and its metabolites. Of note, endoxifen and 4-hydroxy-tamoxifen concentrations were 15.7% and 12.3% higher in the low activity group compared to the high activity group (endoxifen: 31.23 vs. 27.00 nM; 4-hydroxy-tamoxifen: 5.55 vs. 4.94 nM). In Table 2,

**Table 2:** Overview of mean concentration levels and metabolic ratios of tamoxifen, endoxifen, 4-hydroxy-tamoxifen and NDM-tamoxifen by high, medium and low activity groups

	Tamoxifen (nM) (SD)	Endoxifen (nM) (SD)	4-hydroxy-tamoxifen (nM) (SD)	NDM-Tamoxifen (nM) (SD)	MR tamoxifen/NDM-tamoxifen (SD)	MR tamoxifen/4-hydroxy-tamoxifen (SD)	MR 4-hydroxy-tamoxifen/endoxifen (SD)	MR NDM-tamoxifen / endoxifen (SD)
High activity group (*N* = 231)	308.20 (113.17)	27.00 (14.69)	4.94 (2.02)	619.54 (231.10)	0.51 (0.13)	66.82 (23.62)	0.21 (0.09)	32.74 (27.18)
Medium activity group (*N* = 324)	312.12 (128.38)	30.51 (15.66)	5.27 (2.24)	584.42 (224.65)	0.54 (0.14)	63.94 (26.85)	0.19 (0.08)	26.04 (22.25)
Low activity group (*N* = 105)	319.84 (122.13)	31.23 (18.29)	5.55 (2.78)	621.20 (210.66)	0.52 (0.13)	64.52 (31.77)	0.21 (0.10)	30.88 (33.42)
*p* value	0.650	0.016	0.050	0.148	0.027	0.544	0.025	0.010

*SD* standard deviation; *MR* metabolic ratio

an overview of the mean concentration levels and metabolic ratios of tamoxifen, endoxifen, 4-hydroyx-tamoxifen, and NDM-tamoxifen is presented.

### Clinical outcome and 3′-UTR *SULT1A1* groups

**Fig. 3:**
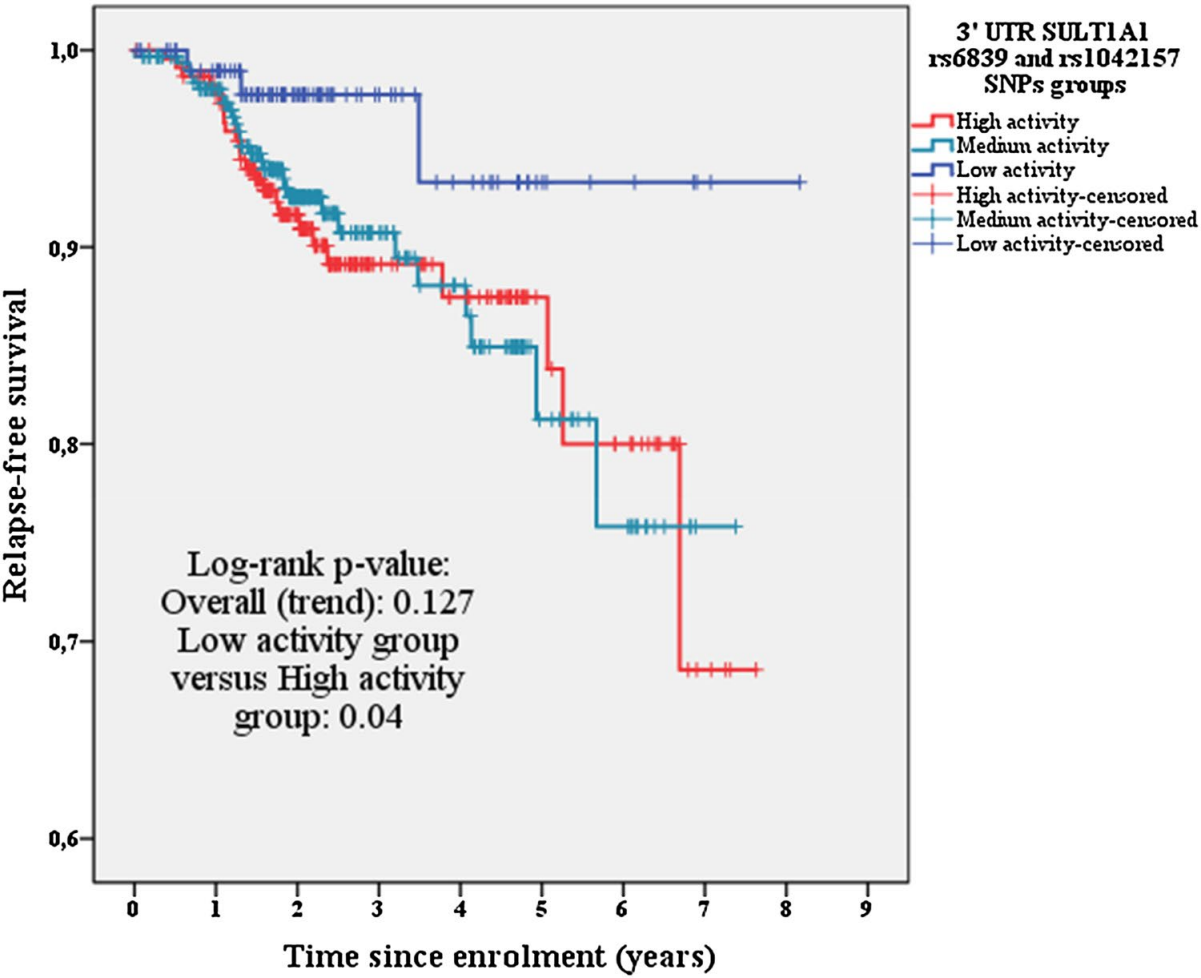
Kaplan–Meier curve comparing. 3′UTR SULT1A1 rs6839 and rs1042157 SNPs groups. 3′-UTR: 3′untranslated region; SULT1A1: Sulfotransferase 1A1

An overall log-rank test comparing the low, medium, and high SULT1A1 activity groups, did not show differences in RFS across the groups, since no statistically significance was obtained (*p* value = 0.127; see Fig. 3). Interestingly, when comparing the low and high activity groups, a statistical difference in RFS was found (Log-rank test: *p* value = 0.042; see Fig. 3).

In the same line, the uni- and multivariable Cox regression analyses also found a trend towards better RFS in the low activity group (Adjusted HR:0.297; 95% CI 0.088–1.000; *p* value: 0.05; see Table 3),

**Table 3:** Cox regression analysis

	Univariable analysis			Multivariable analysis^a^		
	HR	95 % CI	*p* value	HR	95 % CI	*p* value
Age at enrolment (years)	1.017	0.994–1.040	0.146			
Tumor size						
T1	1.000	Reference	(0.316)	1.00	Reference	(0.291)
T2	1.534	0.880–2.657	0.132	1.266	0.722–2.219	0.410
T3/T4	1.419	0.424–4.745	0.570	0.478	0.127–1.804	0.276
Nodal status						
N0	1.000	Reference	(0.053)	1.00	Reference	(0.075)
N1	1.610	0.867–2.968	0.131	1.691	0.897–3.188	0.104
N2	2.388	1.029–5.542	0.043	2.562	1.088–6.030	0.031
N3	3.342	1.230–9.081	0.018	2.898	1.012–8.302	0.048
Grade						
G1	1.000	Reference	(0.420)	1.00	Reference	(0.153)
G2	0.899	0.409–1.977	0.792	0.592	0.261–1.345	0.211
G3	1.330	0.580–3.051	0.500	1.052	0.446–2.483	0.908
HER status						
Negative	1.000	Reference		1.00	Reference	
Positive	1.402	0.634–3.101	0.404	1.771	0.773–4.059	0.177
Histologic classification						
Ductal classification	1.000	Reference	(< 0.001)	1.000	Reference	(< 0.001)
Lobular classification	3.435	1.927–6.121	< 0.001	4.497	2.340–8.643	< 0.001
Others	1.139	0.403–3.222	0.806	1.467	0.509–4.222	0.478
Progesterone status						
Negative	1.000	Reference				
Positive	0.630	0.337–1.175	0.146			
Surgery						
Mastectomy	1.00	Reference				
Breast conserving	0.838	0.491–1.431	0.518			
Surgery axilla						
Sentinel node procedure	1.00	Reference				
Axillary lymph node dissection	1.523	0.879–2.640	0.134			
Chemotherapy						
No	1.000	Reference				
Yes	0.923	0.522–1.630	0.781			
Radiotherapy						
No	1.000	Reference				
Yes	0.793	0.455–1.383	0.414			
Trastuzumab treatment						
No	1.000	Reference				
Yes	1.430	0.646–3.164	0.378			
3′UTR SULT1A1 groups						
High activity group	1.000	Reference	(0.156)	1.000	Reference	(0.131)
Medium activity group	0.939	0.5434–1.622	0.820	0.991	0.564–1.739	0.974
Low activity group	0.310	0.093–1.031	0.056	0.297	0.088-1.000	0.050
3′UTR SULT1A1 groups						
High activity group	1.000	Reference		1.000	Reference	
Low activity group	0.308	0.093–1.022	0.054	0.286	0.084–0.976	0.046

^a^Adjusted for Her2Neu status, histologic grade and classification, tumor size and nodal stage. *3′UTR* 3′ untranslated region

compared to the medium and high activity group. A comparison between the extreme groups, low and high SULT1A1 activity, revealed a significantly lower risk for recurrence in the low activity group in both uni- and multivariable Cox regression analyses (Adjusted HR: 0.286; 95% CI 0.084–0.976; *p* value: 0.046; see Table 3).

### Association of tamoxifen metabolism with rs6839 and rs1042157 SNPs

Genetic variances in CYP2D6 only partly contribute to explaining the inter-patient variability (*R*^2^) of tamoxifen and its metabolites concentrations and metabolic ratios [29, 32]. When rs6839 and rs1042157 SNPs were fitted in the model, the inter-patient variability (*R*^2^) of (log-transformed) concentrations and metabolic ratios of tamoxifen and its metabolites increased for all the cases, by 0.4 to 1.3%. Also, the explained variance (*R*^2^) of the (log-transformed) concentrations of endoxifen only marginally improved from 42.3 to 43.6%. An overview of the rs6839 and rs1042157 covariate analysis is presented in Supplementary Table 2.

## Discussion

This is the first study in which the role of 3′-UTR *SULT1A1* rs6839 and rs1042157 SNPs on tamoxifen metabolism and clinical outcome in early-breast cancer patients was examined. This study shows that patients with low SULT1A1 activity [rs6839 (GG) and rs1042157 (TT)] reached higher endoxifen and 4-hydroxy-tamoxifen concentration levels, but this small effect did not translate in improved RFS.

SULT1A1 is an important enzyme in tamoxifen elimination and it is involved in two relevant parts of the tamoxifen metabolic pathway: the transformation of 4-hydroxy-tamoxifen and endoxifen into 4-hydroxy-tamoxifen sulfate and endoxifen sulfate, respectively. As described by Yu and colleagues, 3′UTR *SULT1A1* rs6839 and rs1042157 SNPs are associated with a decreased SULT1A1 enzymatic activity, and both SNPs contribute to explaining the variability of SULT1A1 enzyme activity [23]. Based on the results of Yu and colleagues, we hypothesized that lower SULT1A1 enzymatic activity conferred by the presence of rs6839 and rs1042157 SNPs would translate in higher concentrations of endoxifen and 4-hydroxy-tamoxifen. Our results confirmed this hypothesis, since higher concentrations of both endoxifen and 4-hydroxy-tamoxifen were found.

The transformation from tamoxifen into NDM-tamoxifen represents 92% of tamoxifen metabolism, while the metabolic conversion from tamoxifen into 4-hydroxy-tamoxifen accounts for only 7% of tamoxifen metabolism [3]. Accordingly, differences in NDM-tamoxifen concentrations would not be as relevant as compared to the other metabolites, whereas small variations in endoxifen and 4-hydroxy-tamoxifen concentrations might be more significant. Our results suggest that the route 4-hydroxy-tamoxifen to endoxifen, might be more important in the presence of a decreased activity of SULT1A1 enzyme, as a consequence of the lower elimination of endoxifen and 4-hydroxy-tamoxifen.

In line with these results, a lower risk for relapse was found in the low activity group, compared to the high activity group. While the increased endoxifen concentration levels and better clinical outcome are completely in line, we feel that this interpretation should be carefully considered, since the association between endoxifen concentration and clinical outcome remains uncertain.

Both endoxifen and 4-hydroxy-tamoxifen have comparable anti-estrogenic activity [4], yet only endoxifen is seen as the most active metabolite of tamoxifen metabolite, since it is found in higher concentrations than 4-hydroxy-tamoxifen [5]. Therefore, the relationship between endoxifen concentration levels and RFS has been investigated, but different ranges for endoxifen concentration have been proposed. For instance, Madelensky et al. described a 26% lower chance of relapse for patients with an endoxifen concentration level above 16 nM (5.97 ng/ml) [33], whereas Helland and colleagues reported an even lower limit of 9 nM (3.36 ng/ml) for better clinical outcomes [34]. In contrast, Neven and colleagues failed to find an association between endoxifen concentration levels and progression-free survival in the metastatic and neoadjuvant setting [35]. In line with these authors, no association between endoxifen concentration and RFS was found in the core CYPTAM study [26, 27]. In the present study, a 15.7% increase of the mean endoxifen serum concentration was found in patients with low SULT1A1 activity, while the explained variance of the concentrations of endoxifen only slightly improved (from 42.3 to 43.6%). Accordingly, the combination of the lack of association between endoxifen concentration and RFS in combination with a barely improved explained variance of endoxifen concentrations, it seems unlikely that there is a true association between *SULT1A1* and RFS caused by the tenue differences in endoxifen concentration levels. Alternative explanations may involve the role of genetic variations in *SULT1A1* in breast cancer risk [36] or in endogenous estrogen metabolism [37].

A potential limitation in our analysis might be the fact that rs6839 was not found in HWE. For the pyrosequencing analysis, quality controls were used, and the call-rate in the samples was above 90%, avoiding therefore any technical problem to be reason for this HWE deviation. Also, we performed the pyrosequencing analysis in isolated DNA from whole blood samples. By this way, we prevented any HWE discrepancy due to potential loss of heterozygosity and HWE using tumor material. The rs6839 genotype frequencies were comparable to those reported in the NCBI database [38]. Another possible weakness in our study might be due to the lack of direct measurement of endoxifen sulfate and 4-hydroxy-tamoxifen sulfate levels; instead, we indirectly assessed effects of the *SULT1A1* SNPs by measuring endoxifen and 4-hydroxy-tamoxifen.

In summary, our results suggest that rs6839 and rs1042157 SNPs have a minor effect on the concentrations and metabolic ratios of tamoxifen and its metabolites, and RFS in women receiving adjuvant tamoxifen, but this impact is not likely to be clinically meaningful.

## Electronic supplementary material

Below is the link to the electronic supplementary material.

Supplementary material 1 (DOCX 15 KB)

Supplementary material 2 (DOCX 14 KB)

## Data Availability

The datasets analyzed during the current study are available from the corresponding author on reasonable request.
