# Supplementary material for: Genetic polymorphisms of 3′-untranslated region of SULT1A1 and their impact on tamoxifen metabolism and efficacy
Source: Breast Cancer Res Treat. 2018 Aug 17;172(2):401–11. doi: 10.1007/s10549-018-4923-7 (PMC6208901; doi:10.1007/s10549-018-4923-7)
Supplement: Supplementary file 1 — Supplementary material 1 (DOCX 15 KB) [file 10549_2018_4923_MOESM1_ESM.docx]

**Table Supplementary 2. Summary of SULT1A1 covariate analysis**

|  |  | **R^2^** |
| --- | --- | --- |
| **Ln Tamoxifen** | **CYP2D6** | 0.003 |
|  | **CYP2D6, rs6839 and rs1042157** | 0.007 |
| **Ln Endoxifen** | **CYP2D6** | 0.423 |
|  | **CYP2D6, rs6839 and rs1042157** | 0.436 |
| **Ln 4-Hydroxy-Tamoxifen** | **CYP2D6** | 0.127 |
|  | **CYP2D6, rs6839 and rs1042157** | 0.138 |
| **Ln NDM-Tamoxifen** | **CYP2D6** | 0.138 |
|  | **CYP2D6, rs6839 and rs1042157** | 0.142 |
| **Ln MR Tamoxifen/NDM-Tamoxifen** | **CYP2D6** | 0.218 |
|  | **CYP2D6, rs6839 and rs1042157** | 0.221 |
| **Ln MR Tamoxifen/4-hydroxy-tamoxifen** | **CYP2D6** | 0.219 |
|  | **CYP2D6, rs6839 and rs1042157** | 0.228 |
| **Ln MR 4-Hydroy-Tamoxifen/Endoxifen** | **CYP2D6** | 0.449 |
|  | **CYP2D6, rs6839 and rs1042157** | 0.453 |
| **Ln MR NDM-Tamoxifen/Endoxifen** | **CYP2D6** | 0.570 |
|  | **CYP2D6, rs6839 and rs1042157** | 0.580 |

MR: Metabolic ratio; Ln Tamoxifen: natural log of Tamoxifen concentration; Ln Endoxifen: natural log of Endoxifen concentration; Ln 4-Hydroxy-Tamoxifen: natural log of 4-Hydroxy-Tamoxifen; Ln NDM-Tamoxifen: N-Desmethyl-Tamoxifen concentration; Ln(MR Tamoxifen/NDM-Tamoxifen): natural log of MR Tamoxifen/NDM-Tamoxifen; Ln(MR Tamoxifen/4-hydroxy-tamoxifen): natural log of MR Tamoxifen/4-hydroxy-tamoxifen; Ln(MR 4-Hydroy-Tamoxifen/Endoxifen): natural log of MR 4-Hydroy-Tamoxifen/Endoxifen; Ln(MR NDM-Tamoxifen/Endoxifen): natural log of MR NDM-Tamoxifen/Endoxifen
