# Supplementary material for: Genetic polymorphisms of 3′-untranslated region of SULT1A1 and their impact on tamoxifen metabolism and efficacy
Source: Breast Cancer Res Treat. 2018 Aug 17;172(2):401–11. doi: 10.1007/s10549-018-4923-7 (PMC6208901; doi:10.1007/s10549-018-4923-7)
Supplement: Supplementary file 2 — Supplementary material 2 (DOCX 14 KB) [file 10549_2018_4923_MOESM2_ESM.docx]

**Supplementary Table 1. Genotype distribution and frequency in the study population**.

| **SNP** |  | **Total individuals (n)** | **Frequency (%)** |
| --- | --- | --- | --- |
| **rs6839** | **AA** | 294 | 44.1 |
|  | **AG** | 260 | 39.0 |
|  | **GG** | 106 | 15.9 |
|  | **Unknown** | 7 | 1.0 |
| **rs1042157** | **CC** | 240 | 36.0 |
|  | **CT** | 298 | 44.7 |
|  | **TT** | 122 | 18.3 |
|  | **Unknown** | 7 | 1.0 |

Unknown: not genotyped or missing data.
